# Surgical management of a delayed post‐traumatic saccular aneurysm of the radial artery

**DOI:** 10.1002/ccr3.4541

**Published:** 2021-07-24

**Authors:** Byron Chalidis, Dimitrios Kitridis, Maria Tirta, Nikiforos Galanis, Panagiotis Givissis

**Affiliations:** ^1^ 1st Orthopaedic Department George Papanikolaou Hospital Aristotle University of Thessaloniki Thessaloniki Greece

**Keywords:** allen test, aneurysm, artery ligation, radial artery, ulnar artery

## Abstract

Post‐traumatic aneurysm of the radial artery is a rare and often misdiagnosed vascular lesion of the wrist. Radial artery ligation can lead to excellent results if Allen test confirmed that ulnar artery is the dominant feeding artery to the hand.

## Graphical abstract

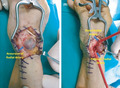

## 1 CASE PRESENTATION

Post‐traumatic aneurysm of the radial artery is an extremely rare vascular lesion and source of pain at the volar‐radial aspect of the wrist. Aneurysm resection and radial artery ligation may lead to excellent outcome if Allen test confirms that ulnar artery is the dominant arterial blood supply to the hand.

**FIGURE 1:**
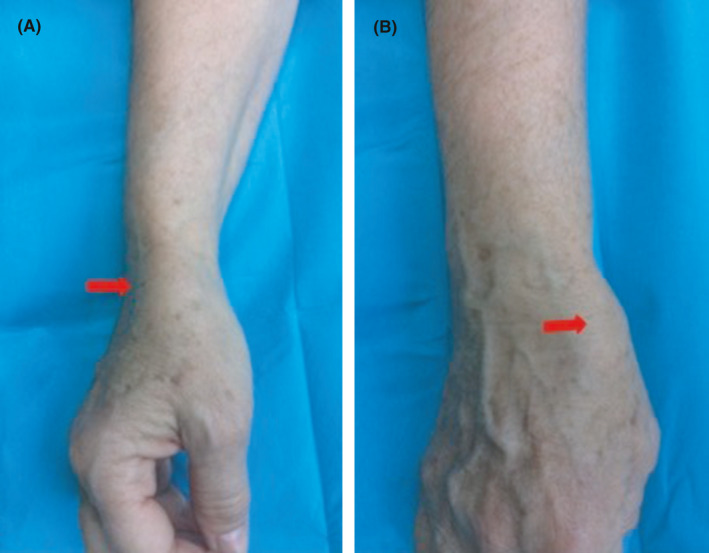
Preoperative images showing the radial A, and dorsal B, view of the soft‐tissue lesion at the anatomical snuffbox of the right wrist

**FIGURE 2:**
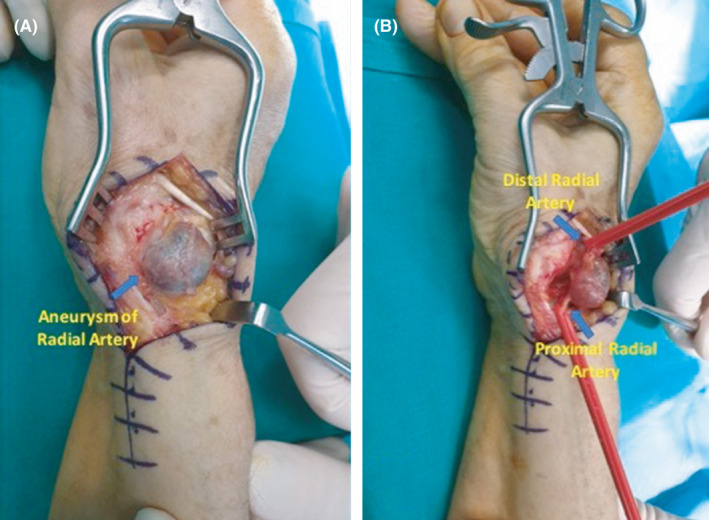
Intraoperative images showing the aneurysm of the radial artery A, and its proximity to proximal and distal part of the artery B

**FIGURE 3:**
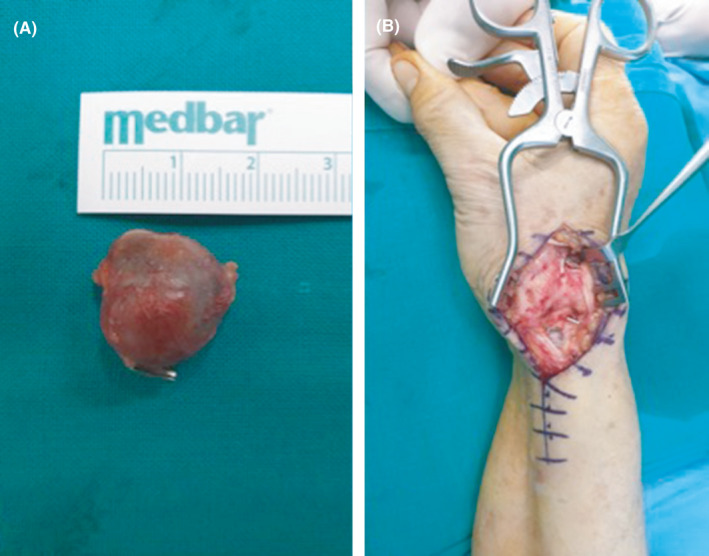
Intraoperative images of the resected aneurysm of the radial artery A, and of the operative field after ligation of proximal and distal parts of the artery B

A 62‐year‐old woman was referred to our department with a painful mass at the anatomical snuffbox of her right wrist that has increased in size the last 5 months. The patient had a history of blunt trauma to the involved wrist approximately 7 years ago. Physical examination revealed a painful, soft, and pulsating tumoral mass (Figure 1). Vascular assessment of both radial and ulnar arteries was taken place with the Allen test that indicated ulnar artery dominance. Furthermore, the Doppler arterial ultrasound displayed a focal dilatation of the right radial artery of approximately 1.8 cm in length. After thorough discussion with the patient regarding the possibility of radial artery ligation, a signed consent form was obtained. Under regional anesthesia, a skin incision was applied over the aneurysm site following the path of the radial artery (Figure 2). The aneurysm sac was excised and the radial artery was ligated proximally and distally (Figure 3). The histopathological report confirmed the nature of the lesion. The postoperative period was uneventful and at the latest follow‐up 1 year after the index procedure, she did not report any pain or disability and she could perform without difficulty all the daily activities.

## 2 DISCUSSION

Aneurysm of the radial artery is a rare post‐traumatic or iatrogenic vascular lesion that can be misdiagnosed as ganglion, synovitis, or soft‐tissue tumor.^1^ Apart from image investigation, evaluation of the radial and ulnar blood flow should be performed preoperatively. After excision of the aneurysmal sac, artery ligation or reconstruction (end to end or graft interposition) may be performed.^1^, ^2^ Resection and radial artery ligation can lead to excellent results if Allen test confirmed the ulnar artery dominancy ^2^.

## CONFLICT OF INTEREST

There are no conflicts of interest associated with this publication, and there has been no financial support for this work that could have influenced its outcome.

## AUTHOR CONTRIBUTIONS

BC drafted and submitted the manuscript. DK obtained the photographs and contributed to the patient care. MT extracted the patient history and assisted in completing edits. NG edited the manuscript. PG contributed to the patient care and critically reviewed the manuscript.

## ETHICAL APPROVAL

Written informed consent was obtained from the patient to publish this case report.

## Data Availability

Data sharing not applicable to this article as no datasets were generated or analyzed during the current study.
